# Free mass distribution of long lasting insecticidal nets lead to high levels of LLIN access and use in Madagascar, 2010: A cross-sectional observational study

**DOI:** 10.1371/journal.pone.0183936

**Published:** 2017-08-29

**Authors:** Alyssa M. Finlay, Jessica Butts, Harilala Ranaivoharimina, Annett H. Cotte, Benjamin Ramarosandratana, Henintsoa Rabarijaona, Luciano Tuseo, Michelle Chang, Jodi Vanden Eng

**Affiliations:** 1 Malaria Branch, Division of Parasitic Diseases and Malaria, U.S. Centers for Disease Control and Prevention, President’s Malaria Initiative, Antananarivo, Madagascar; 2 Malaria Branch, Division of Parasitic Diseases and Malaria, U.S. Centers for Disease Control and Prevention, President’s Malaria Initiative, Atlanta, Georgia, United States of America; 3 National Malaria Control Program, Antananarivo, Madagascar; 4 World Health Organization, Antananarivo, Madagascar; Northwest A&F University, CHINA

## Abstract

**Background:**

Madagascar conducted the first two phases of a national free mass distribution campaign of long-lasting insecticidal nets (LLINs) during a political crisis in 2009 aiming to achieve coverage of two LLINs per household as part of the National Malaria Control Strategy. The campaign targeted households in 19 out of 91 total health districts.

**Methods:**

A community-based cross-sectional household survey using a three-stage cluster sample design was conducted four months post campaign to assess LLIN ownership, access and use. Multivariable logistic regression analysis was used to identify factors associated with household LLIN access and individual LLIN use.

**Results:**

A total of 2211 households were surveyed representing 8867 people. At least one LLIN was present in 93.5% (95% confidence interval [CI], 91.6–95.5%) of households and 74.8% (95% CI, 71.0–78.6%) owned at least two LLINs. Access measured as the proportion of the population that could potentially be covered by household-owned LLINs was 77.2% (77.2% (95% CI, 72.9–81.3%) and LLIN use by all individuals was 84.2% (95% CI, 81.2–87.2%). LLIN use was associated with knowledge of insecticide treated net use to prevent malaria (OR = 3.58, 95% CI, 1.85–6.94), household ownership of more LLINs (OR 2.82, 95% CI 1.85–4.3), presence of children under five (OR = 2.05, 95% CI, 1.67–2.51), having traveled to the distribution point and receiving information about hanging a bednet (OR = 1.56, 95% CI, 1.41–1.74), and having received a post-campaign visit by a community mobilizer (OR = 1.75, 95% CI, 1.26–2.43). Lower LLIN use was associated with increasing household size (OR = 0.81 95% CI 0.77–0.85) and number of sleeping spaces (OR = 0.55, 95% CI, 0.44–0.68).

**Conclusions:**

A large scale free mass LLIN distribution campaign was feasible and effective at achieving high LLIN access and use in Madagascar. Campaign process indicators highlighted potential areas for strengthening implementation to optimize access and equity.

## Introduction

The Global Malaria Action Plan calls for the rapid scale up of key malaria prevention interventions including widespread use of long-lasting insecticidal nets (LLINs) by people at risk for malaria since research has demonstrated their effectiveness in reducing all-cause malaria mortality and morbidity in young children [1, 2]. Monitoring and evaluation of LLIN distribution efforts is important to help determine the impact of LLINs and to inform optimal program implementation strategies within the national malaria control program (NMCP).

In Madagascar, malaria remains a leading cause of morbidity and mortality in children under five years of age. Achieving 80% LLIN coverage in children under five and pregnant women is one of the main targets of the NMCP [3]. Madagascar revised its malaria strategy to rapidly scale up malaria control activities as outlined in the 2008–2012 National Strategic Plan for Malaria Control in view of pre-elimination [3]. Revisions included prioritizing universal coverage with the objective of two LLINs per household to achieve maximum coverage and no longer targeting only pregnant women and children under five. Prior to the strategy revision, LLINs had been distributed when available: 1) free through sub-national integrated immunization campaigns targeting children under five and pregnant women, 2) through sale of highly subsidized LLINs (at approximately $1.50 per LLIN), and 3) through routine distribution during antenatal clinic visits and immunization visits for children under one year old. The combination of these methods contributed to an important increase in ownership of at least one LLIN per household (from 20.5% in 2004 to 79.8% in 2008) as well as an increase in reported LLIN usage among children the previous night (from 13.3% in 2004 to 69.1% in 2008) [4, 5]. However, the gap that remained to cover all households with two LLINs was sizeable; in 2008 the frequency of households owning at least two LLINs was only 27.2% in LLIN-targeted endemic zones [6].

Subsequently the local Madagascar Roll Back Malaria (RBM) partnership with the NMCP/*Service de Lutte Contre le Paludisme*, *(NMCP/SLP*) worked with donors to identify and secure resources to scale up to the nationally-defined coverage goal of two LLINs per household, and maintain target coverage levels through 2012. As a result of the staggered availability of funds and lead-time needed for procurement and delivery of LLINs, Madagascar planned a phased or “rolling” campaign. The overall LLIN campaign and associated goal of two LLINs per household was targeted to 91 of 111 health districts where malaria is endemic per Madagascar’s National Strategic Plan for Malaria Control 2008–2012. The remaining 20 health districts are highland districts with lower malaria transmission and different control strategies including indoor residual spraying (IRS) and epidemic surveillance [3].

Madagascar conducted the first two phases of a stand-alone mass free LLIN distribution campaign in 19 health districts on the East Coast, a geographic zone with an estimated population of 3.8 million, during November 16–22 (12 districts) and December 14–20, 2009 (7 districts). Community volunteers known as community “mobilizers” were recruited to visit each household prior to the campaign, and provided two LLIN vouchers per household, LLIN education, and information about the planned distribution dates and sites. LLINs were transported and distributed to the population free-of-cost at community-level distribution points in order to reach even the most remote areas. Household representatives were asked to bring their vouchers to the distribution point to exchange for two LLINs on a pre-planned distribution day. Over 1,730,000 LLINs were distributed. After the campaign, the community mobilizers were asked to visit households to deliver educational messages and encourage and assist hang-up of newly distributed LLINs. The campaign was led by the Madagascar Ministry of Health and Family Planning as a collaborative effort among the Global Fund to Fight AIDS, Tuberculosis and Malaria (GFATM); Population Services International; UNICEF; the U.S. President’s Malaria Initiative; the International Federation of Red Cross and Red Crescent Societies; the Malagasy Red Cross; USAID; U.S. Centers for Disease Control and Prevention (CDC); World Health Organization; and others.

The distribution of LLINs through a community-level free mass distribution was expected to increase the national levels of LLIN ownership and usage in Madagascar by 2010. A household survey provided an opportunity to quickly assess coverage results and LLIN use after the early roll-out phases. The main objectives of the survey were to measure household LLIN coverage, access, and use in the 19 districts which were part of the first two phases of the universal coverage LLIN campaign.

## Materials and methods

### Location and timing of survey

**Fig 1:**
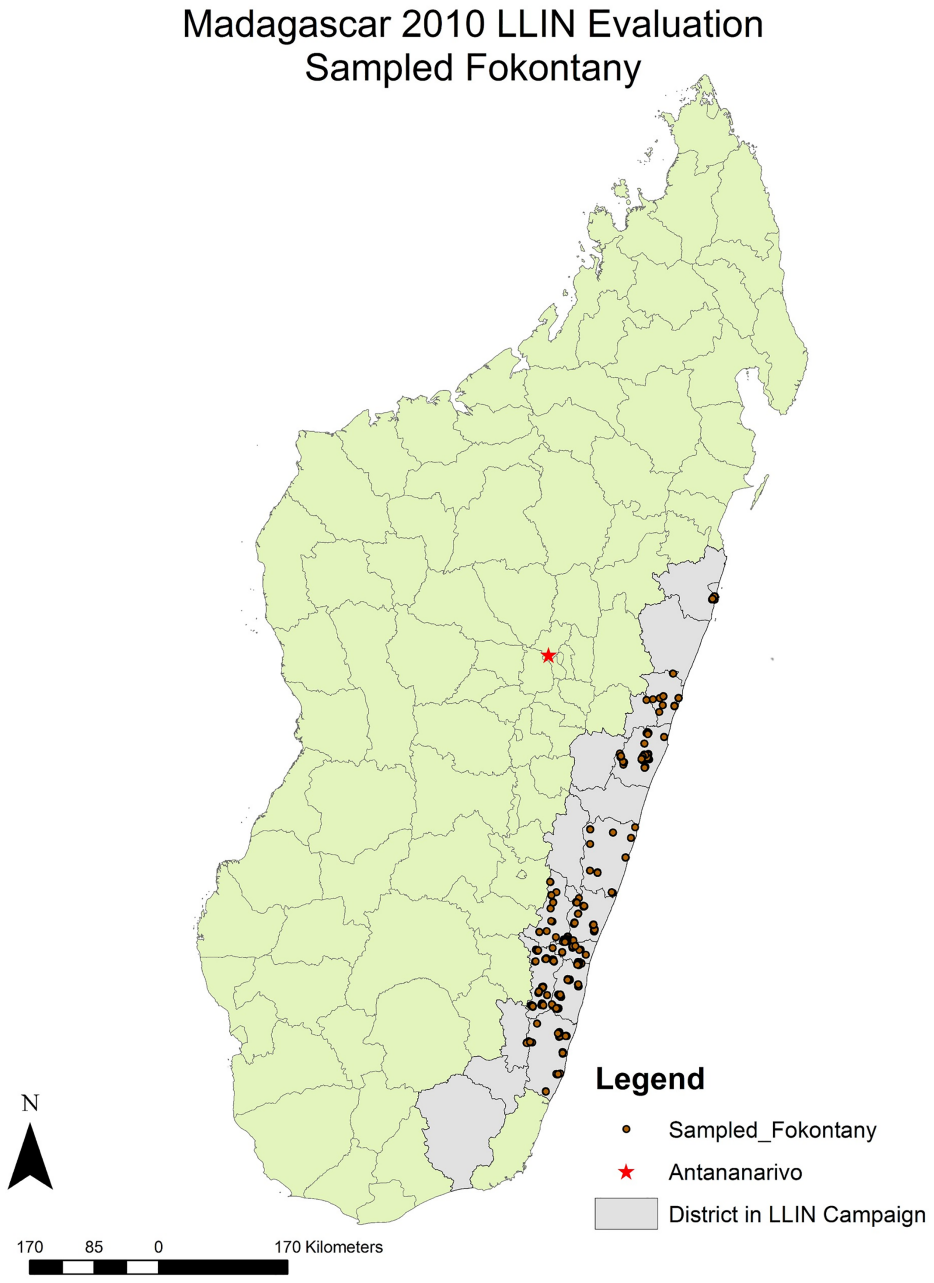
Map of Madagascar districts involved in the 2009 LLIN distribution campaign and sampled fokontany (villages) included in the evaluation.

The community-based cross-sectional household survey took place between April and May 2010, during the end of the rainy season on the east coast of Madagascar. The survey was sub-national, focusing in 4 regions and 19 districts which were targeted in the first two phases of the mass free LLIN distribution campaign (Fig 1).

### Sampling and data collection

Three-stage cluster sampling was conducted. Ten districts were selected using probability proportional to population size (PPS) sampling. Population estimates were based on official district estimates extrapolated from the most recent national census in 1993 which estimated the population of Madagascar to be 19.7 million in 2008. Within each of the districts, 9 fokontany (villages) or enumeration areas were selected by PPS. All households within the selected enumeration areas were then mapped using personal digital assistant (PDA)-based GPS technology (custom software, CDC, Atlanta GA) and twenty-five households were selected for interviews by random sampling using the household listing created by the GPS mapping exercise [7].

Survey teams administered a detailed questionnaire to each household following a standard format similar to those used LLIN coverage surveys elsewhere [8–10]. Household members, bednets, and sleeping spaces were inventoried. Household-level questions focused on bednet ownership; type, quantity and quality of nets; net use and net hang up; campaign-specific process indicators such as: how many nets they received and retained as part of the mass campaign distribution; information, education and communication (IEC); household visits by community mobilizers; and whether they received a voucher. Demographic characteristics of household members and reported bednet use were collected. A household was defined as “all persons eating from the same food pot”, in line with the definition used in previous surveys in Madagascar.

### Data analysis

Data from the Microsoft^®^ Access database were transferred to Stata^®^ version 10 (Stata Corporation, College Station, Texas, USA) and SAS version 9.3 (SAS Institute, Cary, NC) for verification, data management, and analysis. Specialized analysis survey procedures using Stata^®^ (SVY procedure) or SAS (Proc Survey) were used to produce valid estimates and calculate standard errors to account for clustering at the primary sampling unit (district) level and sampling weights. Descriptive statistical analysis and Rao-Scott chi-square statistics were used for tests of association. Two multivariable logistic regression models were constructed to assess relationships between independent explanatory variables and key outcomes: 1) household LLIN ownership of at least one LLIN per two people, and 2) LLIN use the night preceding the survey by individuals (residing in households owning at least one LLIN), while controlling for confounding factors. For the household ownership model, in order to get the odds of each individual in a household having access to a LLIN, the outcome “access” was modeled using the single-trial specification syntax in SAS with one record corresponding to each person in the household and using binary coding for whether they had access to a LLIN based on the RBM goal of 1 net: 2 person ratio and the number of LLINs owned by the household. Pre-existing LLIN estimates were calculated by taking total LLINs per household at the time of the survey and subtracting the number of campaign LLINs received and present [10]. A map of Madagascar indicating districts included in the 2009 distribution campaign and the fokontany sampled in the evaluation was generated using ESRI (ArcGIS) software.

A section of the questionnaire addressed the overall economic status of housing and living quarters. These questions were adapted from the 2000 Multiple Indicator Cluster Survey and 2003–4 and 2008 Demographic and Health Surveys (DHS) and were used to develop a wealth index using principal components analysis [11]. Questions included type of materials used in construction of the housing dwelling, ownership of certain assets, and water sources. A wealth index score was calculated for each household and based on this score, households were assigned to one of five wealth quintiles from least wealthy (1) to most wealthy (5) using quintile cut-off points established for the 2008 DHS. Economic equity ratio was calculated as the ratio of proportion in the richest to the poorest wealth quintile.

For the purposes of this paper, net ownership and use results are presented for LLINs instead of the standard insecticide treated net (ITN) indicators because the difference between ITNs and LLINs in this setting was negligible (< 1%). The most recent RBM Monitoring and Evaluation Reference Group indicators were used to assess universal access to and utilization of LLINs: 1) the proportion of households with a sufficient number of LLINs to protect all individuals sleeping in the household assuming each LLIN is shared by two people, 2) the proportion of the population with access to a LLIN in the household (i.e., universal coverage defined as one LLIN per two people), and 3) the proportion of the population that slept under a LLIN the previous night.[12]

### Ethical considerations

The evaluation protocol and questionnaire were reviewed by the Madagascar Ministry of Health Ethics Review Board and the CDC Ethical Review Committee. The evaluation was approved by both entities. There were no known risks involved with this evaluation and participation was voluntary with informed consent. Verbal consent was approved by Ethics boards and obtained prior to collecting survey data after explaining the objective of the study and types of questions the participants would be asked, the advantages of participating, confidentiality of responses and emphasizing the voluntary nature of participating. Verbal consent was documented using the PDA.

## Results

### Household characteristics

Among the 90 fokontany sampled, there were a total of 2239 households selected. Among these, 2211 (98.8%) were surveyed, including 54.3% from districts covered in the November campaign and 45.7% from districts covered in the December campaign. There were 1005 households with at least one child under the age of five and 240 households with at least one pregnant woman among household members (see Table 1). The average household size was 4.1 people (95% CI, 3.9–4.3 persons) and 41.6% of households were among the two poorest wealth quintiles. Selected household assets are listed in Table 1.

**Table 1:** Household, individual and bed net characteristics.

Household Characteristics	Number	Weighted%* (95% CI^†^)
Households surveyed	2211	-
Households in districts participating in November 2009 campaign	1536	54.3 (26.7–81.9)
Households in districts participating in December 2009 campaign	675	45.7 (18.1–73.3)
Individuals living in surveyed household	8867	-
Average number of members per household	4.1	(3.9–4.3)
Range of household sizes	1–18	-
Households with children under 5 years old	1005	44.0 (38.5–49.5)
Households with pregnant women (15–49 years old)	240	9.6 (7.6–11.5)
Sleeping spaces inventoried	3719	-
**Households by Wealth Quintile**		
1 (poorest)	433	17.5 (9.7–25.4)
2	654	24.1 (14.9–33.2)
3	546	20.4 (12.1–28.6)
4	309	14.5 (12.4–16.6)
5 (richest)	269	23.5 (1.0–46.0)
**Selected Household Assets**		
Type of roof		
Thatch, palm or leaf	1447	61.2 (42.3–80.2)
Rustic mat, palm/bamboo	192	6.1 (3.5–8.8)
Sheet metal	446	26.6 (9.1–44.2)
Type of wall		
Bamboo/cane/palm	1617	68.7 (55.0–82.5)
Wood planks	251	15.3 (4.4–26.2)
Owns a		
Radio	924	48.0 (35.4–60.6)
TV	239	20.6 (1.8–39.3)
Cell phone	339	25.9 (4.1–47.7)
Bicycle	306	21.5 (7.9–35.0)
Motorcycle	64	5.1 (0.3–10.4)
Car	20	1.8 (0.01–3.6)
Livestock or other animals	1400	57.3 (46.0–70.0)
Malaria prevention activities cited by household		
Sleeps under bed net	1942	89.3 (85.4–93.2)
Keeps the environment clean	325	20.7 (8.1–33.3)
Removes stagnant water around the house	190	11.1 (3.9–18.4)
Closes the windows	198	9.0 (7.2–10.9)
Burns herbs or dung	218	8.2 (4.5–11.9)
Uses insecticide coils	116	6.2 (3.0–9.4)
Covers with blankets or sheets at night	200	7.8 (3.8–11.8)
Takes medication (nivaquine, etc.)	13	0.4 (0.2–0.6)
Knowledge of advantages of using a LLIN^‡^ as cited by household		
Protection against mosquitos	1445	65.4 (60.5–70.1)
Peaceful sleep	1101	49.7 (44.2–55.3)
Prevent malaria	1384	62.6 (58.5–66.7)
Privacy	39	1.8 (1.2–2.3)
Don’t know	53	2.4 (1.5–3.3)
**Individual Characteristics**		
Total individuals	8867	-
Male gender	4228	47.2 (46.3–48.1)
Children < 5 years old	1372	14.8 (12.9–16.6)
Women (15–49 years old, not pregnant)	2012	23.6 (21.8–25.4)
Pregnant women (15–49 years old)	243	2.4 (1.9–2.9)
Slept in the house the night before	8716	98.4 (97.6–99.1)
Education Level		
No formal education	3240	33.5 (24.5–42.6)
Primary school	4113	43.4 (38.0–48.8)
Middle school	903	13.3 (7.2–19.4)
Secondary school	339	5.9 (0.2–38.7)
Higher than secondary school	110	2.0 (0.2–3.9)
Don’t know/not sure	162	1.8 (1.2–2.4)
Slept in household the night prior to the survey	8716	98.4 (97.6–99.1)
Average number of people per sleeping space	3.4	(3.3–3.5)
Range of number of people per sleeping space	1–15	-
**Bed net Characteristics**		
Total bed nets inventoried (any bed net)	4551	-
LLINs	4278	94.0 (92.2–95.7)
Proportion of existing LLINs used the previous night	3078	73.8 (69.6–78.0)
Bed net hanging the night before the survey	3183	72.0 (67.3–76.8)
Old bed nets, not hanging and no longer used for sleeping	228	4.4 (2.8–6.0)
Old LLINs, not hanging and no longer used for sleeping	140	3.0 (2.0–4.1)
Bed nets in usable condition	4323	95.6 (94.0–97.2)
Ever-treated bed net (ITN^§^)	4140	95.3 (93.8–96.9)
LLIN	4138	95.3 (93.7–96.9)
Campaign net	3540	77.7 (68.1–87.4)
Campaign LLIN	3488	76.3 (67.3–85.3)
Source from campaign—free	3363	74.9 (65.6–84.2)
Source from campaign–paid something	177	2.8 (0.8–4.9)
Other source—free	184	5.3 (3.4–7.1)
Other source–paid something	599	17.0 (9.1–24.9)
New in good condition (no holes)	3419	73.8 (69.5–78.0)
New still in original packaging	353	8.1 (5.8–10.5)
Average number of bed nets per household	2.1	(2.0–2.2)
Range of bed nets per household	1–8	
Average number of sleepers per LLIN used	2.6	(2.4–2.7)
Range of sleepers per LLIN used	1–10	-

*Adjusted for clustering and sampling weights

^†^Confidence Intervals (CI)

^‡^Long lasting insecticidal net (LLIN)

^§^Insecticide treated net (ITN)

When households were asked what their members do for protection against malaria, 89.3% cited sleeping under a bednet, 20.7% cited keeping the environment around their home clean, and 11.1% cited draining stagnant water. The most frequently reported advantages of using a LLIN were: protection against mosquitos (65.4%), protection against malaria (62.6%), and peaceful sleep (49.7%).

### Individual characteristics

Among the 8867 persons living in the surveyed households, 4228 (47.2%) were male, 1372 (14.8%) were children under five years old, and 243 (2.4%) were pregnant women. Almost all individuals, 8716 (98.4%) reported sleeping in the house the night prior to the survey. The majority of individuals, 64.6%, had a primary school education or higher.

### Bednet characteristics

There were a total of 4551 bednets inventoried and 3719 sleeping spaces counted among the households surveyed. There was an average of 2.1 bednets per household (95% CI, 2.0–2.2), ranging between 1–8 bednets per household. The average number of individuals sharing a sleeping space was 3.4 (95% CI, 3.3–3.5) ranging from 1 to 15 people.

Among the 4551 bednets, 4280 met the criteria of an ITN and the remaining 271 were identified as untreated nets. All of the ITNs were LLINs except two (4278, >99.9%). The overall number and proportion of LLINs that were reported to be used the previous night was 3078 or 73.8%. Among LLINs used, there was an average of 2.6 (95% CI, 2.4–2.7) people sleeping under the LLIN the previous night (Table 1). Households reported that 228 (4.4%) of the nets inventoried were no longer used for sleeping because they were perceived by users as too old, including 140 LLINs. Further details of net characteristics can be found in Table 1.

### LLIN campaign

#### Progress towards bednet campaign coverage target of two LLINs per household

Campaign process indicators are summarized in Table 2. Only 70.1% (95% CI, 59.0–81.2%) of households reported receiving the campaign goal of at least two LLINs, and 11.1% of households reported that they did not receive any LLINs at all during the campaign. Results were fairly equitable across wealth quintiles. The top five reasons for not receiving a campaign net were: household members were absent during the distribution (28.1%), household members were not listed on the distribution census list (12.2%), no bednets were available (14.0%), household members did not have a voucher (14.2%), and household members did not possess an official identity card (4.6%).

**Table 2:** Campaign process indicators.

Campaign Indicator	All Households, N = 2211	
	Number	Weighted %* (95% CI^†^)
Number of nets that each HH^‡^ received from the campaign		
Two or more nets	1634	70.1 (59.0–81.2)
One net	346	18.8 (9.6–28.0)
Zero nets	231	11.0 (7.2–15.0)
HH received information about the campaign	2019	91.8 (89.6–94.0)
HH received a pre-distribution visit by a mobilizer	1494	66.1 (60.9–71.3)
**HH received a voucher**	1823	84.2 (81.0–87.5)
Mobilizer explained how to use voucher	1665	91.0 (86.9–95.1)
Number of vouchers received:		
1 voucher	716	32.5 (28.3–36.8)
≥2 vouchers	1107	51.7 (45.7–57.7)
Did not receive a voucher	361	14.9 (11.7–18.1)
Don’t know	27	0.8 (0.6–11.6)
**HH traveled to distribution point**	1918	86.9 (84.2–89.6)
Time to travel to distribution point:		
Less than 30 minutes	1197	68.2 (58.1–78.2)
Between 30 minutes and one hour	333	16.2 (10.6–21.8)
One to two hours	194	7.5 (4.3–10.7)
More than 2 hours	130	5.4 (2.6–8.3)
Half a day	40	1.6 (0.6–2.7)
One day	16	0.8 (0.0–1.6)
More than one day	1	0.02 (0.0–0.1)
Don’t know	7	0.2 (0.0–0.5)
Type of information messages received at distribution point		
How to hang the net	870	40.9 (26.3–55.5)
How to wash/take care of the net	896	42.3 (28.5–56.2)
Why it is important to use the net	721	35.1 (29.2–40.8)
Other	114	6.0 (4.3–7.7)
**HH received a post-distribution visit by mobilizer**	577	26.9 (21.3–32.4)
Received a hang-up demonstration	277	40.6 (24.0–57.2)
Told importance of sleeping under bed net every night	471	81.7 (77.2–86.2)
Told how to care for the bed net	261	40.2 (25.7–54.8)
Received other malaria information	43	6.9 (1.6–12.2)
Did not receive any IEC messages during visit	75	14.3 (5.7–22.9)

*Adjusted for clustering and sampling weights

^†^Confidence Intervals

^‡^HH: Household

At the time of the survey, households were asked if they still possessed the campaign LLINs that they initially received. Among the 1980 households that received one or more campaign LLINs, 1848 (93.7%) reported they had retained all the campaign nets and 1965 (99.3%) retained at least one campaign net. There were a total of 132 campaign nets that were no longer in possession by the households. For the majority of nets (84% of the 132 not retained) the reason cited was because they were given away to others.

### Campaign mobilization, information, education and communication

Among the 2211 households responding to the survey, 2019 (91.8%) had received information about the bednet campaign. The primary source of information most commonly cited was: 1) the fokontany chief (60.2%), 2) a community mobilizer (21.8%), and 3) a health worker (11.1%). Campaign Information Education Communication (IEC) materials and messaging were less commonly cited: only 1.1% reported the radio as a source, 0.4% reported banner/posters, 0.1% reported Tam Tam (village criers), and 0.3% reported television as their main source of information.

Two thirds of households (66.1%) reported they received a pre-distribution visit by a campaign community mobilizer. Only 1823 households (84.2%) said they received vouchers prior to the campaign: 32.5% of households received only one voucher and 51.7% received two or more vouchers (Table 2). The large majority (91.0%) of households that received vouchers reported the community mobilizer explained how to use the voucher. Among the 361 households that did not receive a voucher, 17.4% said there were not enough vouchers left, 14.9% said they were not visited by a mobilizer, and only 6 (1.3%) reported they did not get a voucher because they did not have enough money to pay for it although vouchers were supposed to be distributed for free. Almost half (42.1%) did not know why they were not given a voucher.

Among the 1918 households (86.9%) that traveled to the distribution point, 98.9% traveled on foot and 83.5% felt the distance was reasonable. Over two-thirds of households (68.2%), reported it took less than 30 minutes of travel time to reach the distribution point from their home, 16.2% said it took between 30 minutes and 1 hour, and 16.3% said it took over 1 hour of travel time (Table 2). Only 17 households (1%) said it took one full day or more of travel time to reach the distribution point. When households were asked what type of information they received at the distribution point, 40.9% cited how to hang a net, 42.3% said they received messages on washing and caring for nets, and 35.1% were told why it was important to use the net.

Only 577 (26.9%) of households received a visit by a mobilizer after the campaign (Table 2). Among these, 277 (40.6%) households received a hang-up demonstration and the reported delivery of key post-campaign messaging was variable: 81.7% reported they were told the importance of sleeping under the net every night and only 40.2% were told how to care for their net. Furthermore, 14.3% of households said they did not receive any education or malaria prevention messages during the post-campaign visit.

### LLIN ownership and LLIN access

**Fig 2:**
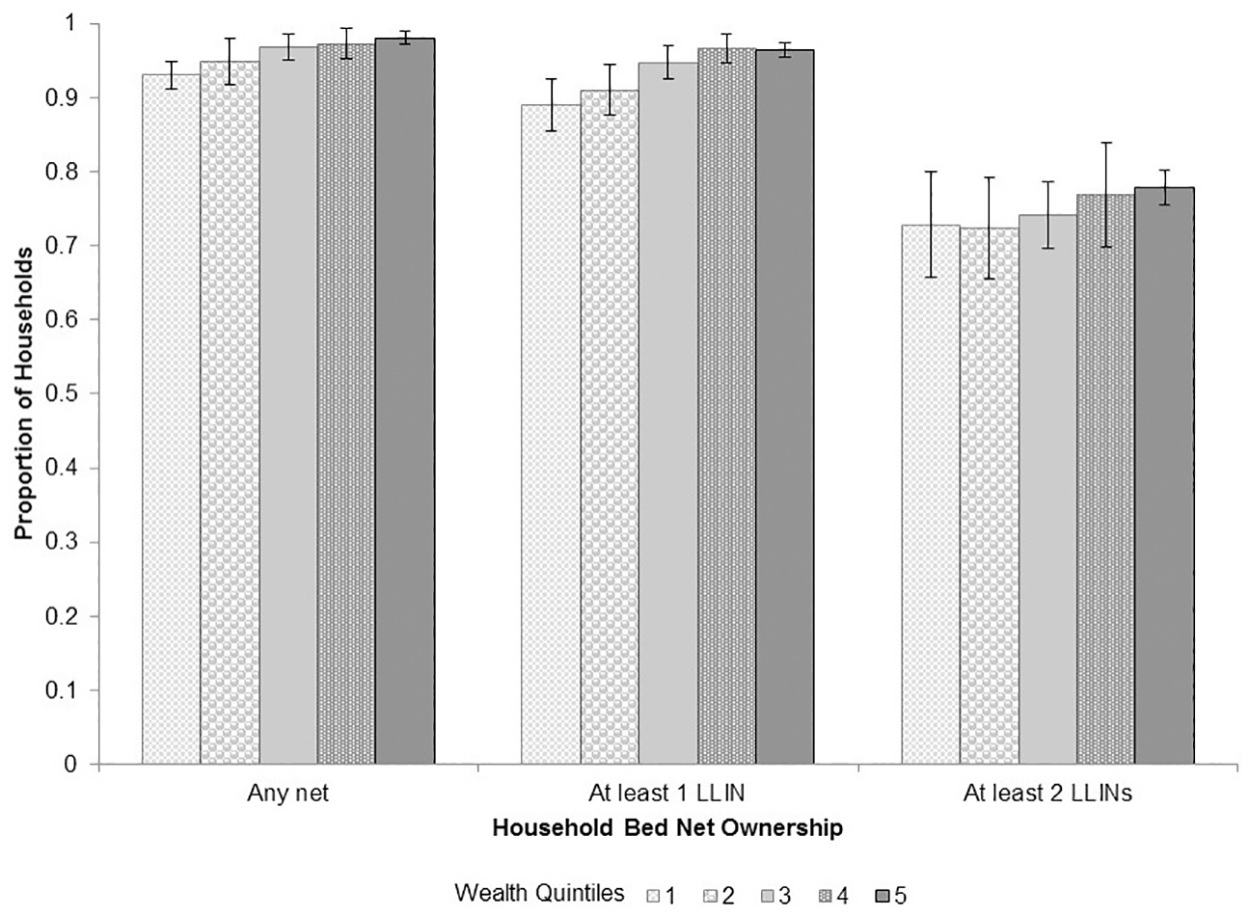
Proportion of households owning 1) any net 2) at least one LLIN and 3) at least two LLINs, by wealth quintile (1 = poorest, 5 = wealthiest quintile).

Among the 2211 households responding to the survey, post-campaign net ownership was high: 93.5% (95% CI, 91.6–95.5%) owned at least one LLIN, and 74.8% (95% CI, 71.0–78.5%) owned at least two LLINs. Fig 2 shows household LLIN ownership by wealth quintile: 89.1% (95% CI, 85.6–92.6%) of households in the poorest wealth quintile owned at least one LLIN compared to >96% in the two richest wealth quintiles; however, for ownership of at least two LLINs, differences between wealth quintiles were not significant. The average number of LLINs per household was 2.0 (95% CI, 1.9–2.1).

There were 1005 households with at least one child under five years old. Net ownership in this subgroup was equally high: 93.8% (95% CI, 90.8–96.8%) of households with at least one child under five owned at least one LLIN and 77.5% (95% CI, 73.8–81.2%) owned at least two. Among the 240 households with at least one pregnant woman, the percentage owning one LLIN and two LLINs were similar at 93.9 (95% CI, 90.2–97.7%) and 72.8 (95% CI, 65.0–80.6%), respectively

**Fig 3:**
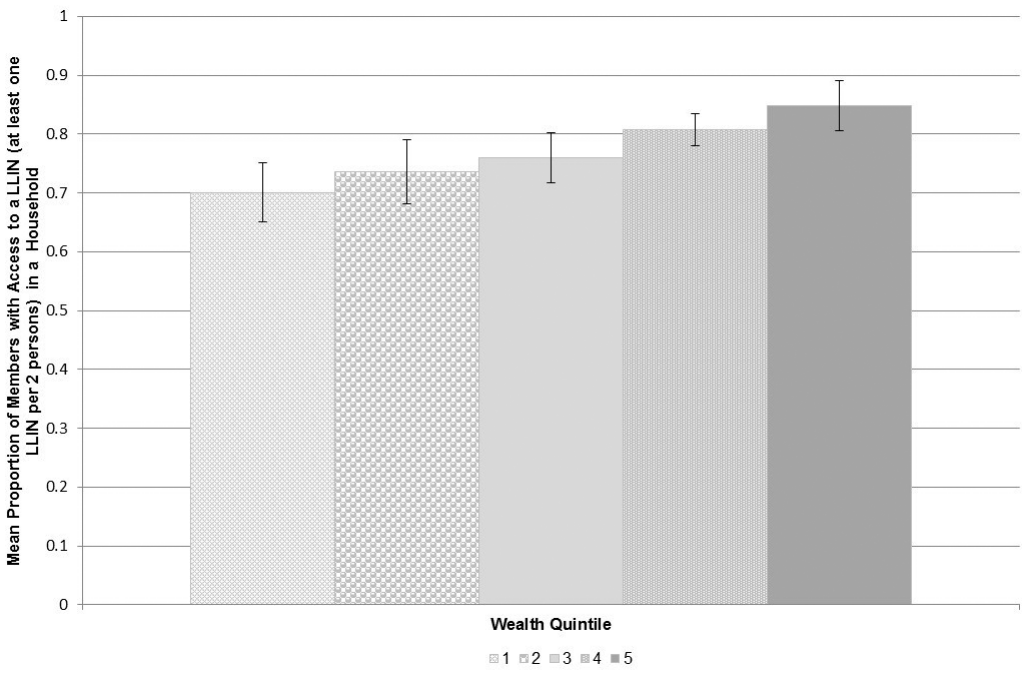
Average proportion of household members with access to a LLIN (at least one LLIN per 2 persons) by wealth quintile (1 = poorest, 5 = wealthiest quintile).

Among all households, 58.6% (95% CI, 53.3–63.9%) owned enough nets to meet the RBM goal ratio of at least one LLIN for every two people. Access measured as the proportion of the population that could potentially be covered by household-owned LLINs was 77.2% (77.2% (95% CI, 72.9–81.3%). Fig 3 illustrates this indicator stratified by wealth quintile showing that the proportion of the population with access to a LLIN increased with increasing wealth status. The equity ratio of the wealthiest to the poorest households was 1.2 (wealthiest quintile = 84.9% (95% CI, 83.3–86.4%), poorest quintile = 70.1% (95% CI, 65.1–75.1%)). Reasons reported for not receiving a bednet during the campaign included the following: households not registered, no bednets available at the distribution point, and individuals did not have an identification card to present.

In contrast, pre-existing LLIN coverage was low at 31.1% (95% CI, 19.1–43.2%), 9.2% (95% CI, 3.2–15.2%), and 10.2% (95% CI, 6.0–14.4%) for the proportion of households owning of at least one pre-existing LLIN, at least two pre-existing LLINs, and at least one pre-existing LLIN per two people, respectively.

A multivariable model to assess factors associated with LLIN access defined as a minimum of one LLIN per two people (RBM goal) was created and odds ratios associated with LLIN access are shown in Table 3. The final model consisted of 24 variables. Decreased odds of an individual having access to a LLIN in a household was significantly associated with having a child under five years old in the household (OR 0.58, 95% CI 0.50–0.67), poor wealth status (OR 0.36, 95%CI 0.24–0.54 for the poorest quintile compared to the richest), not owning a radio (OR 0.75, 95% CI 0.63–0.90), more sleeping spaces in the household (OR 0.77, 95% CI 0.69–0.86), not receiving a pre-campaign voucher (OR 0.4, 95% CI 0.33–0.49), and living in a district covered by the December campaign vs. districts covered in the November campaign (OR 0.75, 95% CI 0.63–0.90). Campaign factors significantly associated with increased LLIN access included having received a pre-campaign household visit (OR 1.2, 95% CI 1.01–1.42). Households with a member that knew malaria could be prevented by sleeping under a LLIN were associated with increased odds of access (OR 2.33, 95% CI 1.88–2.90). LLIN access was not associated with presence of a pregnant woman in the household, highest level of education in the household, knowledge that LLINs protect against mosquito bites or having received any information about the campaign. Nor was it associated with travel time or relative distance from the distribution point to the household.

**Table 3:** Factors associated with household access to LLINs (ratio of LLINs to people).

Household Access to LLINs*	Unadjusted OR^†^	*p*	Adjusted OR^†^	95% CI^‡^	*p*
Household with at least one pregnant woman	0.93	0.62	1.10	0.86–1.42	0.44
At least one child under five years old in household	0.68	<0.0001	0.58	0.50–0.67	<0.0001
Number of people sleeping in household	0.78	<0.0001	-	-	-
Highest level of education in household					
No formal education	Ref	Ref	Ref	Ref	0.32
Primary school	1.19	0.002	1.12	0.88–1.44	
Middle school	1.54	0.94	1.27	0.92–1.75	
Secondary school	2.39	0.003	1.54	1.03–2.29	
Higher than secondary school	2.06	0.23	1.31	0.70–2.44	
Wealth quintile					
1 (poorest)	0.42	<0.0001	0.36	0.24–0.54	<0.0001
2	0.50	0.006	0.43	0.29–0.64	
3	0.58	0.32	0.44	0.30–0.64	
4	0.78	0.032	0.60	0.41–0.87	
5 (richest)	Ref	Ref	Ref	Ref	
Campaign wave					
November 2009	Ref		Ref	Ref	<0.01
December 2009	0.82	0.027	0.75	0.63–0.90	
Cited malaria could be prevented by sleeping under an insecticide treated mosquito net	2.68	<0.0001	2.33	1.88–2.90	<0.0001
Cited most important advantage of LLIN was to protect against mosquito bites	1.28	0.009	1.11	0.95–1.30	0.18
Received information about the campaign	2.03	<0.0001	1.13	0.86–1.49	0.38
Received a pre-campaign household visit	1.56	<0.0001	1.20	1.01–1.42	0.04
Received a campaign voucher					
Yes	Ref	Ref	Ref	Ref	<0.0001
No	0.33	<0.0001	0.40	0.33–0.49	
Don’t know	1.09	0.021	1.51	0.80–2.85	
Possession of a radio	1.14	0.14	0.75	0.63–0.90	<0.01
Number of sleeping spaces in household	0.96	0.51	0.77	0.69–0.86	<0.0001
Number of people sharing a sleeping space in household	0.68	<0.0001	-	-	-
Traveled to distribution point and travel time					
≤ 1 hour	Ref	Ref	Ref	Ref	0.55
> 1 hour	0.69	0.37	1.07	0.86–1.32	
Don’t Know	0.38	<0.0001	0.63	0.23–1.75	
Distribution point location relative to household					
In same village	Ref	-	Ref	Ref	0.22
In another village in same fokontany	0.76	0.039	0.85	0.69–1.04	
In another village outside fokontany	0.64	0.97	0.81	0.65–1.00	
Other	0.35	<0.0001	0.94	0.35–2.53	

*Long lasting Instecticidal Nets (LLINs).

^†^Odds Ratio (OR).

^‡^Confidence Interval (CI).

### LLIN use

**Fig 4:**
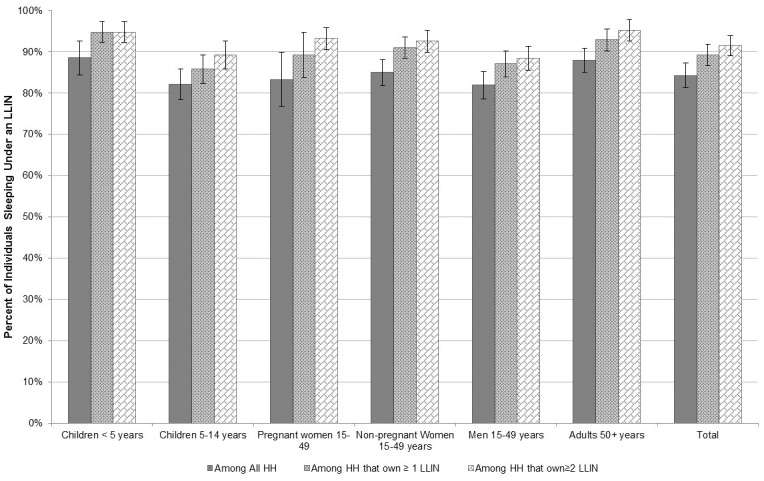
LLIN use by specific age group and gender among 1) all households 2) households that own at least one LLIN and 3) households that own at least two LLINs.

The majority, 84.2% (95% CI, 81.2–87.2%) of individuals that slept in the household the night before the survey reported using a LLIN. Among vulnerable groups providing information about LLIN use, including 1372 children under five and 243 pregnant women, reported LLIN use was 88.5% (95% CI, 84.4–92.6%) and 83.3% (95% CI, 76.8–89.8%), respectively. Reported LLIN use was high (>80%) among all age and gender groups including children 5–14 years old, non-pregnant women 15–49 years old, men 15–49 years old and adults over 50 years old illustrated in Fig 4. The proportion of the population reporting having slept under a LLIN increased for each of these groups in the subset of households that owned at least one LLIN and increased further among households that owned at least two LLINs. Among the 1634 (70.1%) households that owned at least two LLINs, the proportion of all individuals, children under 5 and pregnant women that reported sleeping under a LLIN was very high at over 90% as shown in Fig 4.

Universal LLIN use, defined as all household members sleeping under a LLIN, was estimated by calculating the proportion of households in which every member reported sleeping under a LLIN the night before the survey. Among the 2198 households with at least one person that slept in the household the night before the survey, 76.8% (95% CI, 73.2–80.4%) reported that all household members slept under a LLIN, with equity across wealth quintiles. When these households were further stratified by those that owned at least one LLIN and those that owned at least two LLINs, universal LLIN use was even higher at 82.1% (95% CI, 78.6–85.6%) and 85.2% (95% CI, 81.8–88.6%), respectively.

**Fig 5:**
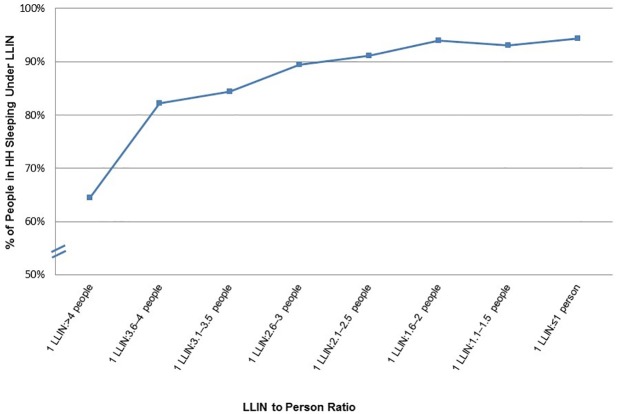
Proportion of people sleeping under a LLIN within households by LLIN to person ratio.

LLIN use was stratified by the LLIN-to-person ratio, shown in Fig 5. LLIN use was over 90% when the LLIN-to-person ratio was 1:3 (0.33) or more. Among households that achieved the RBM goal of at least one LLIN per two persons (ratio 1:2 or more, ≥0.5), 93–94% of members were using a LLIN. In contrast, in households where there were more than four people per LLIN, use fell to ≤ 65%.

Only 3183 (72%) of bednets were reported to be hanging the night before the survey. Of the 1140 usable nets that were not hanging, the main reasons cited were: all household members were already sleeping under other bednets (37.1%), the net was too old and worn (1.9%), the net was used for travelers or guests (17.5%), the net was being saved for future use (9.9%), and there was not enough room to hang the net (9.7%). There were rare reports of bednets not hung because they were being used for other purposes (0.6%) or not hung because they were perceived to not work or because household members did not like to use them (0.5%).

Results of a multivariable analysis to identify factors associated with the odds of individuals using a LLIN, among households owning LLINs, are shown in Table 4. Increased odds of individuals using a LLIN was associated with older age (OR 1.02, 95% CI 1.01–1.02) and inversely associated with the head of the household having a primary education or higher (OR 0.74, 95% CI 0.66–0.83). Increased odds of use was also associated with the following household characteristics: lower economic status or wealth quintile (OR 1.84, 95% CI 0.88–3.85 for the poorest compared to the richest), number of children under five in the household (OR 2.05, 95% CI 1.67–2.51), knowledge by a member of the household that malaria is prevented by sleeping under a LLIN (OR 3.58, 95%CI 1.69–6.94), living in a household that traveled to and received information on how to hang a bednet at the distribution site (OR 1.56, 95% CI 1.41–1.74), and living in a household that received a post campaign visit by a community mobilizer who told them to sleep under the net nightly (OR 1.75, 95% CI 1.26–2.43). Higher odds of LLIN use was inversely associated with household size (OR 0.81, 95% CI 0.77–0.85). Increased odds of LLIN use was also independently associated with living in a household with higher quality wall material such as earth, bamboo reinforced with mud, wood planks compared to a simple palm/cane/bamboo wall. LLIN use was not associated with gender, household ownership of a radio or type of household roof.

**Table 4:** Factors associated with LLIN use.

LLIN* Use	Unadjusted OR†	*p*	Adjusted OR†	95% CI‡	*p*
Female gender	1.14	0.01	1.10	0.99–1.23	0.08
Age	1.01	<0.0001	1.02	1.01–1.02	<0.0001
Primary education or higher	0.69	0.01	0.74	0.66–0.83	<0.0001
Wealth Quintile					
1 (poorest)	1.57	0.05	1.84	0.88–3.85	<0.0001
2	1.46	0.09	1.45	0.70–3.00	
3	1.22	0.68	1.28	0.70–2.32	
4	0.76	0.01	0.78	0.67–0.91	
5 (richest)	Ref	-	Ref		
Household size (number of people sleeping in household)	0.89	0.0006	0.81	0.77–0.85	<0.0001
Number of children under five years old in household	1.58	0.006	2.05	1.67–2.51	<0.0001
Number of LLINs owned by household	1.68	0.003	2.82	1.85–4.3	<0.0001
Number of free nets in household	1.29	0.008	-	-	-
Number of nets from the campaign in household	1.61	<0.0001	-	-	-
Number of hanging LLINs in household	7.34	<0.0001	-	-	-
Number of sleeping spaces in individual’s household	0.69	0.0007	0.55	0.44–0.68	<0.0001
Number of people sharing an individual’s sleeping space	1.06	0.53	-	-	-
Knowledge in household that malaria is prevented by sleeping under an insecticide treated net	2.97	0.001	3.58	1.85–6.94	0.0002
Live in HH that traveled to distribution site	1.08	0.61			
Lives in HH that traveled to distribution site and received information on how to hang a bed net	1.82	<0.0001	1.56	1.41–1.74	<0.0001
Lives in HH that traveled to distribution site and received information on importance of bed net usage	1.12	0.35			
Lives in HH that received a post-campaign visit	1.17	0.17	-	-	-
Lives in HH that received a post-campaign visit and					
Volunteer said to sleep under the net nightly	1.42	0.0006	1.75	1.26–2.43	<0.0001
Volunteer did not mention sleeping under net	0.60	<0.0001	1.34	0.98–1.82	
Lives in HH that did not receive a post-campaign visit	Ref	Ref	Ref	Ref	
Live in HH that received a post-campaign visit and was shown how to hang net	1.51	0.01	-	-	-
Live in HH that received a post-campaign visit and was told importance of sleeping under bed net	2.37	0.0001	-	-	-
Household roof material					
Thatch	Ref	Ref	Ref	Ref	0.43
Sod	1.33	0.01	1.39	0.66–2.93	
Mat	0.81	0.84	0.74	0.36–1.53	
Sheet Metal	0.78	0.51	0.94	0.50–1.76	
Other	0.62	0.18	1.00	0.38–2.63	
Household wall material					
Palm/cane/bamboo	Ref	Ref	Ref	Ref	<0.0001
Dirt/earth	0.61	0.001	1.14	0.63–2.04	
Bamboo with mud	1.93	0.003	1.70	0.82–3.53	
Wood planks	0.85	0.19	1.29	1.05–1.59	
Other	0.92	0.66	1.67	1.03–2.71	
Possession of a radio	0.83	0.32	1.41	0.92–2.16	0.11

*Long lasting Instecticidal Nets (LLINs).

^†^Odds Ratio (OR).

^‡^Confidence Interval (CI).

## Discussion

The 2009 LLIN campaign was the first free mass distribution campaign targeting all individuals (i.e., not limited to pregnant women and children under 5) and attained historic high rates of both LLIN access and use in Madagascar. The campaign reduced disparities in ownership and use across economic groups, improving equity, which was consistent with results of free mass distribution campaigns in other countries [13–16]. The large majority of nets used by household members were in good physical condition and thus, if used correctly, would provide both individual protection against malaria as well as community-level reductions in mosquito density and survival to reduce malaria transmission exposure [17].

### Performance against the targets

Insecticide-treated mosquito nets were first distributed in Madagascar in 2002 through social marketing and starting in 2005, through free routine distribution in health clinics targeting pregnant women and children [18]. An integrated campaign which distributed both free LLINs on a large scale for the first time during a national campaign to provide measles vaccination, mebendazole, and vitamin A to children under five resulted in household ownership of at least one LLIN of 76.8% and LLIN use by pregnant women, children under 5, and all household members in the targeted districts at 68.5%, 80.8% and 59.9%, respectively [19]. Compared to these previous efforts, the stand-alone 2009 free universal mass distribution campaign increased LLIN ownership by >30% and LLIN use by >25% (see S1 Table). Furthermore, using the pre-existing LLIN coverage estimate as a baseline, the campaign increased household ownership of at least two LLINs by 68% and increased household ownership of at least one LLIN per two people from 10.2%, immediately pre-campaign, to 58.6% post-campaign, representing more than a fivefold increase, moving towards the RBM goal.

The average number of LLINs owned per household was two overall, meeting the national strategy target; however, only 70.1% of households reported receiving two or more campaign LLINs during the 2009 distribution and it remains concerning that 13% of households were not reached at all, highlighting distribution weaknesses. Campaign supervisors reported distribution sites that ran out of nets or had to further ration nets during the campaign indicating that several sites did not have enough to provide two LLINs for every household in their communities. After the campaign, compiled data from the household registration process revealed that the registered population of the 19 districts was 116% of that projected from the adjusted census estimates that were used to plan and quantify nets needed for the campaign, which corroborates the theory that there were insufficient nets and could further explain why the campaign goal of two LLINs per household was not met.

Analysis using 2013 MERG indicators measuring access to LLINs based on the recommended ratio of one LLIN for every two persons reveals the fixed “two nets per household” distribution approach during the campaign fell short of the RBM goal of one LLIN for every two persons. The fixed distribution methodology of two LLINs per household, regardless of size, is likely to have led to inequitable distribution of nets given the variation in household size. The mean household size in the survey was 4.1 with a wide range and 36% of households had more than four members sleeping in the household indicating that two LLINs would provide suboptimal coverage in this group. LLIN use was over 90% among households with a LLIN to person ratio of 1:≤3 (0.33), moreover, the average number of people reported sleeping under each LLIN was 2.6. Thus, in order to ensure all household members are adequately protected in Madagascar one LLIN for every two persons is needed.

### Lessons learned from campaign planning and implementation

Campaign implementation process indicators identified key weaknesses in the household registration and voucher distribution process, including charging “hidden fees” for vouchers or nets when they were meant to be free. Vouchers were expensive, logistically difficult to distribute, and sub-optimally used. This resulted in some households not receiving any vouchers and a substantial portion only receiving one voucher, causing an inequitable and insufficient distribution of nets. Campaign monitors anecdotally reported instances where vouchers were falsely reproduced in the field threatening the integrity of the campaign. Vouchers posed a similar barrier during an integrated vaccination/bednet campaign in Niger and vouchers schemes used to distribute nets to pregnant women attending antenatal clinic services have common operational challenges [15, 20, 21]. Vouchers can work well in some settings [10]–however given the implementation challenges in Madagascar and the significant added expense to produce and distribute them, it is difficult to justify future use.

Among households that did not receive a campaign net, the majority did not receive a pre-campaign visit by a mobilizer underscoring the need for a more fair and transparent pre-campaign registration process. Households that received a pre-campaign visit were independently associated with higher LLIN access. Few households had post-campaign visits by community mobilizers and for those that did, visits were of poor quality (e.g., IEC messages not delivered adequately, less than 50% received hang-up demonstrations).

The main source of communication about the campaign cited by households was the fokontany chief, in addition to the mobilizers and health center staff. This important community leader role should be integrated effectively in future campaign planning to optimize information dissemination. Sites appeared to be well placed as most households reported that the distance they were required to travel was reasonable. Over 80% of households said travel to the distribution point took less than one hour and travel times were not found to be associated with lower LLIN access or use thus the campaign organization of one distribution site per three fokontany was adequate. This is an important consideration for future campaigns given the increased cost associated with each additional distribution point. One caveat is that the population density is high on the East coast and the organization of one distribution point per three fokontany may not work equally as well in other parts of the country where distances between fokontany are much larger and fokontany may be more remote and physically isolated.

### Factors associated with LLIN access and use

There are limited reports of factors associated with high intra-household access to a LLIN (at least one LLIN per two people). When considering household ownership of at least one LLIN, determinants of coverage included factors similar to those reported in this survey. Higher ownership associated with increased malaria knowledge was reported in a sub-district in Madagascar in 2008 prior to large scale campaign distribution, and has been reported in other countries [22, 23]. Households citing malaria could be prevented by sleeping under an ITN were positively associated with both better access and higher use in our setting.

The proportion of the population using a LLIN (84.2%) was higher than population LLIN access, supporting the hypothesis that there is a strong culture of LLIN acceptance in Madagascar and there are minimal barriers to use when nets are available in contrast to other settings where LLIN distribution led to increased ownership but did not result in a concomitant increase in use as expected [24–32].

The strongest predictor of individual LLIN use was having more LLINs available in the household. Using survey data from 15 countries, Eisele et al. showed that ITN use by children under five years old increased as intra-household ITN access increased [30]. Recent surveys from Nigeria and Sierra Leone also documented improved household access was associated with higher LLIN use by all individuals, similar to our findings in Madagascar [31, 33]. To improve coverage in future campaigns, quantification estimates and allocation of LLINs to households should be based on household size as recommended by Kilian et al. [34].

Lack of ownership was the predominant reason that members of these poorer households were not protected. Reasons reported for not receiving a bednet during the campaign point to modifiable deficiencies in the campaign implementation process. Recommendations were subsequently made to include non-registered households and persons without identification if they were verified by local authorities. Among wealthier households the largest proportion of non-users lived in households owning and hanging at least one LLIN suggesting other factors are likely to explain this gap–such as inadequate quantity of nets to cover all household members, deficiency in knowledge of the importance of using a bednet, or other reasons inhibiting users from sleeping under a bednet. Pulford et al. conducted a review of the literature among published household surveys and qualitative studies of reasons for not using a mosquito net when available and found the most widely reported reasons were discomfort from heat and perceived low mosquito density [35]. However, these were the least frequently cited reasons for not using nets (<4%) in this Madagascar survey.

Households that received a post campaign visit were also significantly associated with use indicating that targeted post-campaign interpersonal communication activities could effectively promote use, although the evaluation was not designed to test this hypothesis. Evidence of the association between behavior change communication strategies (BCC) and behaviors like net use has been mixed. A recent cluster randomized trial in Togo demonstrated an association between post-campaign BCC and net use [36]. In Ethiopia, repeated malaria IEC interventions with heads of households at the community level have resulted in behavior change and increased LLIN use [37]. Keating et. al. reported an association between high ITN use and exposure to malaria-related messages in communities in Zambia with near universal coverage, although a specific intervention to provide IEC at the household level using community health workers in itself did not significantly influence ITN use [38, 39]. The effectiveness of community level IEC/BCC efforts may be country or community specific given a similar cluster-randomized trial of post-campaign household visits and targeted IEC among intervention communities in Uganda also did not show an increase in LLIN use compared to control communities [40].

In our setting, 8.2% of LLINs were found new and untouched in their original packaging and almost 1/3 of nets not hung were being reserved for guests or future use. Effective mobilization should aim to educate household members on the importance of using the new (more effective) LLINs with an emphasis on replacing older nets that are still hanging. Retention and use of older nets in poor condition, even after acquiring new LLINs, has been reported in other settings [39]. Overall, very few LLINs were repurposed or misused, a finding similar to that recently reported in Zambia and Sierra Leone, and one that is contrary to popular belief [33, 39, 41].

Multiple studies report households having a member with a higher education and literacy level have been associated with increased LLIN use, similar to our findings, and this may facilitate understanding of malaria prevention health promotion messages and behavior [22, 27, 42–46]. In Madagascar, malaria prevention messages had been disseminated through mass media during off-campaign years, as well as through campaign-specific mass media, primarily radio. This could explain why owning a radio was associated with higher LLIN access. Radio possession was also identified to be an important factor associated with higher LLIN use in Ethiopia [47] and listening to a radio once a week was reported as a determinant of use among pregnant women in a recent review [22]. Although it is not possible to discern which specific activities may have been the most effective in promoting malaria prevention behaviors from this survey, developing effective BCC activities, especially targeting less educated households, should be a priority even in the setting of a well-established net culture such as in the East Coast of Madagascar.

Multivariable logistic regression analysis of factors associated with LLIN access confirmed the association between increasing wealth and better access. A similar finding was reported by Thwing et al. in Senegal where poorer households had high ownership of at least one net, but intra-household access was higher among wealthier households [10]. Although LLIN access inequity between rich and poor has vastly improved, some disparities remain between socio-economic groups and continue to be a program challenge [19, 39, 43, 48, 49]. In contrast, increased LLIN use was independently associated with poorer households compared to wealthier households, a finding similar to that reported for children under five years old after an integrated free distribution campaign in Madagascar in 2007 [50, 51]. While there have been several reports of higher ITN use among wealthier populations compared to poorer ones [43, 52, 53], analysis of a few large household surveys have reported higher ITN use among those living in households in the poorest wealth quintile similar to our findings, especially when analyzing the use of bednets distributed free of cost [22, 40, 43, 51].

Surprisingly, households with at least one child under five were associated with lower LLIN access despite a long-standing history of LLIN distribution BCC and strategies that targeted vulnerable groups. Given the low coverage of pre-existing LLINs it is possible that previous LLINs distributed to target the under five year old children were already more than three years old and had become either unusable with regular wear and tear, were discarded or given away. Despite this result for access, individuals living in households with more children under five were associated with higher LLIN use; this finding has been reported previously in a small study from a sub-district of Madagascar, as well as other settings, and could be a result of higher perceived vulnerability to malaria by small children leading to higher overall LLIN use in these households [43, 54]. It is likely program IEC/BCC efforts from previous antenatal clinic LLIN distribution efforts and social marketing in Madagascar also reinforced the use of LLINs in households with children under 5 years old, especially on the East Coast where it was these were the main methods of distributing LLINs from 2006–2009. The number of sleeping spaces in the household were inversely associated with LLIN use and warrants further analysis of intra-household sleeping and net use patterns to potentially inform both distribution strategies and BCC activities to promote optimal use [55].

These survey results reflect the success of the first two phases of a three-phase national campaign and lessons learned were used to inform subsequent LLIN campaign planning. The 2009 campaign and the subsequent 2010 LLIN distribution campaign covering additional geographic areas increased LLIN ownership to 94% among at risk malaria zones nationwide, access to 77% and use among the general population to 82% by May 2011, resulting in almost half of the estimated 34,000 lives saved among children under five between 2000 and 2011 [18]. Large scale-up of net ownership through free mass distribution campaigns is feasible and effective at achieving high ownership and retention six months after distribution and high use in Madagascar by all individuals as well as vulnerable groups. Improvements in campaign planning and implementation could further ensure equitable LLIN access.

The 2011 MIS results represented relative immediate post-campaign coverage and use resulting from both the 2009 LLIN campaign (East Coast) and the subsequent 2010 LLIN campaign (remaining malaria at risk areas in the North, West and South). However, later studies showed high coverage and use rates were not sustainable over time. Kesteman et al. conducted a cross sectional survey in 2012, over two years after the 2009/2010 campaign and found LLIN ownership to be as low as 40–65% depending on the geographic area, access was only 43% and use 64% [56]. The 2013 MIS reported areas of the county that did not benefit from additional campaign distributions in the interim had declines in ownership of at least one LLIN to 68%, access to 39% and use to 51% compared to those that did (ownership of at least one LLIN 96% and use 87%) [57]. These repeated cross-sectional measures over time underscore the challenges and limitations on the ground of maintaining adequate malaria prevention using existing LLIN technology under program conditions in Madagascar.

### Limitations

Self-reported household ownership and use, specifically sleeping under a net the night before the survey, was not verified by observation, and is a limitation of the standard household survey methodology. The survey teams were only able to visually confirm the presence of 82% of reported LLINs in the households. However, we believe people would be more likely to under-report ownership in the hopes of being given another net thus LLIN ownership could be slightly underreported. Also there were 52 bednets that were identified as campaign nets but could not be verified to be LLINs per standard survey methods described above. It is possible these nets were also LLINs which would slightly increase LLIN ownership and use estimates.

Survey data were collected six months after the campaign and self-reported information on voucher receipt and exchange, household visits by community mobilizers and other campaign-type indicators could be subject to recall or reporting bias; however, we would expect this to be minimal. Furthermore, some key indicators like in-home post-campaign hang-up demonstrations are relatively unusual and distinct events in the community that are unlikely to be subject to recall bias.

In addition this cross-sectional survey was not able to directly assess LLIN use over time which has been reported to deteriorate in other settings.[43] Longitudinal studies are needed to address this important programmatic question.

## Supporting information

S1 Table Comparison of key ITN ownership and use indicators between households surveys on the east coast 2008–10, Madagascar. (TIF)
