# Supplementary figures and images for: Free mass distribution of long lasting insecticidal nets lead to high levels of LLIN access and use in Madagascar, 2010: A cross-sectional observational study
Source: PLoS One. 2017 Aug 29;12(8):e0183936. doi: 10.1371/journal.pone.0183936 (PMC5574546; doi:10.1371/journal.pone.0183936)

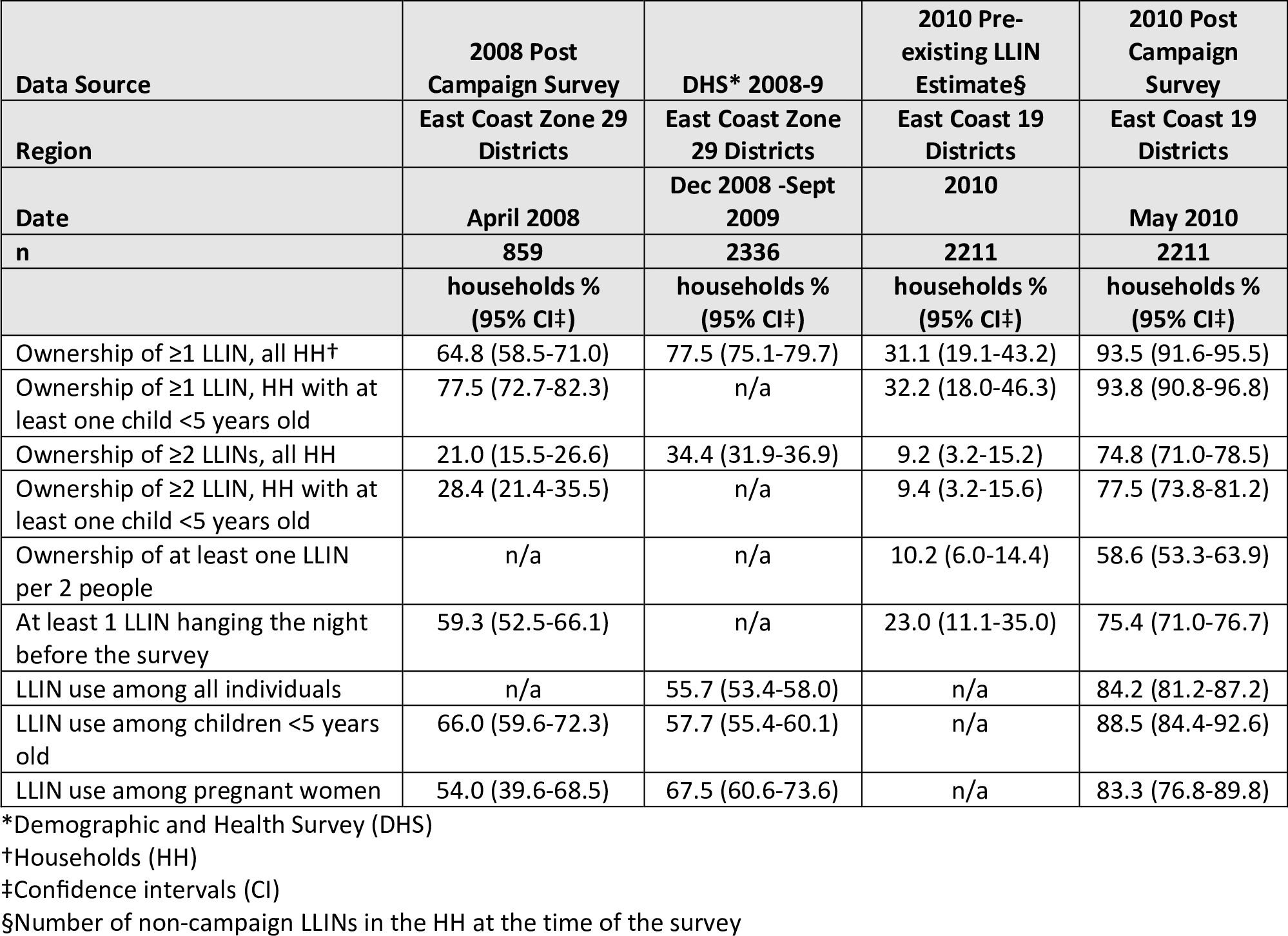

Supplement: S1 Table — (TIF) [file pone.0183936.s001.tif]
